# Comparative analysis of clinical efficacy of stereotactic robot-guided puncture hematoma drainage and conventional puncture hematoma drainage in the treatment of intracerebral hemorrhage

**DOI:** 10.12669/pjms.40.8.8833

**Published:** 2024-09

**Authors:** Xin Gong, Hai-qing Dong, Xin Li, Zhen-jie Liu

**Affiliations:** 1 Xin Gong, Department of Neurosurgery, Baoding No.1 Central Hospital, Baoding, 071000, Hebei, China; 2 Hai-qing Dong, Department of Neurosurgery, Baoding No.1 Central Hospital, Baoding, 071000, Hebei, China; 3 Xin Li, Department of Neurosurgery, Baoding No.1 Central Hospital, Baoding, 071000, Hebei, China; 4 Zhen-jie Liu, Department of Neurosurgery, Baoding No.1 Central Hospital, Baoding, 071000, Hebei, China

**Keywords:** Robot-guided, stereotactic, Puncture hematoma drainage, Intracerebral hemorrhage, Clinical efficacy

## Abstract

**Objective:**

To compare and analyze the clinical effectiveness of conventional puncture hematoma drainage and stereotactic robot-guided puncture hematoma drainage in managing intracerebral hemorrhage.

**Methods:**

This is clinical comparative research. One hundred and twenty patients with the intracerebral hemorrhage who underwent puncture hematoma drainage in Baoding No.1 Central Hospital from March 2020 to May 2023 were included and were assigned into the control groups(n=60) and experimental groups(n=60) according to different treatment methods. The experimental group underwent stereotactic robot-guided puncture hematoma drainage, while the control group underwent conventional puncture hematoma drainage treatment. The duration and situation of surgery, levels of inflammatory factors, as well as preoperative and 1-week postoperative GCS scores and NIHSS scores were compared and analyzed between the two groups.

**Results:**

In comparison with the control group, the experimental group exhibited considerably less surgical duration(p=0.00), higher amount of intraoperative blood drainage and hematoma clearance rate(p=0.00). The experimental group possessed a substantially more reduced incidence of complications(10%) in comparison with the control group(25%), with a statistically substantial distinction(p=0.03). After therapy, CRP, TNF-a, and IL-6 degrees were considerably more decreased (p=0.00) in the experimental group in comparison with the control group, while GCS grades were considerably more prominent and NIHSS grades were considerably more reduced (p=0.00).

**Conclusion:**

Stereotactic robot-guided puncture hematoma drainage is a dependable and safe operative method to treat patients who had intracerebral hemorrhage, resulting in various benefits such as short length of operation, less injury, less inflammatory reaction, high hematoma clear efficiency and satisfactory recovery of neurological function.

## INTRODUCTION

Intracerebral hemorrhage, as one of the most severe complications of hypertension, is most commonly found in the basal ganglia, thalamus and putamen.^1^ With the acute onset and high disability rate, intracerebral hemorrhage is characterized by typical signs such as hemiplegia, hemianesthesia and hemianopsia. In case of a massive hemorrhage, it will easily lead to consciousness disorder and seriously endanger the life and health of patients.^2^ In most cases, patients with intracerebral hemorrhage are in need of an emergency operation. The outcome of the operation is not only related to the patients’ functional recovery, but also to their lives and the medical and social burden on their families.

At present, the preferred surgical approaches for treating such patients include craniotomy for hematoma removal, puncture hematoma drainage, and minimally invasive hematoma removal. Among them, craniotomy for hematoma removal is more traumatic and less tolerable to patients, with a narrower range of indications, poorer prognosis and high cost.^3^ In the context of witnessing the continuous advancement of minimally invasive concepts in recent years, minimally invasive and minimally invasive techniques have become the mainstream surgical modality in teating hypertensive intracerebral hemorrhage. Most Chinese scholars^4^ adopt the described surgical techniques, which are characterized by stereotactic puncture hematoma aspiration. This method is highly safe and yields excellent prognostic outcomes by effectively removing the hematoma and promoting neurological function improvement.^5^

Nevertheless, this method performs puncture manually with reduced puncture accuracy and has certain drawbacks. Neurosurgery robots, in recent years, have been used in the clinic. Doctors can provide a novel treatment method for intracerebral hemorrhage with the help of robots, taking advantage of their minimally invasive characteristics, precision, high safety and selection of optimal puncture path.^6^ Specifically, neurosurgical robots, with the outstanding advantage of being suitable for performing high-precision operations, have brought about a series of technological changes in terms of precise positioning, minimal injury and quality of operation. Based on the development trend of pursuing safety, minimally invasive and precision in neurosurgery, robots have also incomparable good results in minimally invasive treatment on this basis.^7^

In this study, the efficacy of two operation methods, stereotactic robot-guided puncture hematoma drainage and conventional puncture hematoma drainage, in the treatment of intracerebral hemorrhage was compared and analyzed to clarify the merits and drawbacks of the two methods, with a view to choosing the appropriate operation for clinical treatment of intracerebral hemorrhage.

## METHODS

This is clinical comparative research. One hundred and twenty patients with the intracerebral hemorrhage who underwent puncture hematoma drainage in Baoding No.1 Central Hospital from March 2020 to May 2023 were included. They were assigned into the control groups(n=60) and experimental groups(n=60) according to different treatment methods. The sample size required for each group was calculated by the formula:


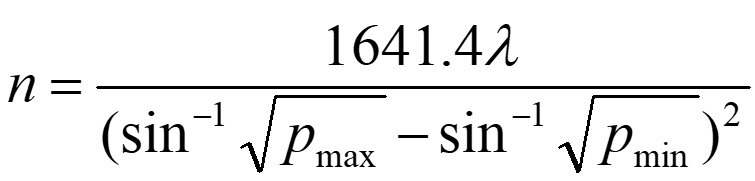


Stereotactic robot-guided puncture hematoma drainage was administered to patients in the experimental group, while conventional puncture hematoma drainage was used to treat patients in the control group.

### Ethical Approval:

The research was authorized by the Institutional Ethics Committee of Baoding No.1 Central Hospital (NO.:[2023]082;May 3,2023), written informed consent was provided by all the individuals participating in the study.

### Inclusion criteria:

- Patients who are eligible the diagnostic standard of intracerebral hemorrhage and require surgical intervention.^8^
- Patients whose interval from onset to treatment was <48 h.
- Patients without serious changes in cardiopulmonary function before admission.
- Patients with the first onset of the disease.
- Patients whose clinical information was complete.
- Patients whose individuals and their families were willing and able to cooperate to complete the study and had good treatment compliance.
- Patients who provided informed consent.

### Exclusion criteria:

- Individuals who have intracranial bleeding caused by other vascular diseases including arteriovenous malformations, cerebral aneurysms, intracranial aneurysms, trauma, or coagulopathy resulting from prolonged use of anticoagulant and antiplatelet medications
- Patients whose clinical information are incomplete.
- Patients with organ diseases or functional failure.
- Patients with unstable vital signs who cannot withstand treatment.
- Individuals who cannot participate in the study due to other reasons.

### Conventional puncture and trepanation-drainage:

According to the CT film of the patient’s head, the target point was identified as the center point of the largest level of hematoma, which was determined at the corresponding scalp projection position with a special positioning ruler and marked. The skin at the puncture point was cut longitudinally about 1.0 cm, and the skull drill was used to drill holes at the target site. After the drill bit had an obvious bone breakthrough feeling, the directional skull drill was removed, the bone chips were cleaned, and a keyhole device was placed for fixation. The dura puncture needle punctured the dura mater, and the brain puncture needle punctured the hematoma along the direction of the keyhole device. Once the hematoma cavity was identified, a special dura dilator was used to expand the dura mater, and then the drainage tube was carefully inserted into the hematoma cavity through the puncture channel. The drainage tube skin moved down two cm and the drainage tube was fixed, and the scalp was sutured all over.

### Surgical robot trepanation and drainage:

On the day of operation, body surface markers were attached, and the head CT scan was performed with a thickness of 1mm, which was stored in the robot computer after the scan was completed. The surgical robot computer planned the surgical target and path. The support arm was connected to the operating vehicle and the three-nail head frame. Clicked [Surgical positioning]-[Robotic arm positioning], and selected the “Verification target” path. The robotic arm automatically moved to the verification point, and the observation and positioning accuracy was within 2 mm. The surgical area of the head was disinfected and covered with sterile towels.

The robotic arm was sleeved with a sterile bag, and a sterile guide and an adapter were installed. The robotic arm automatically moved to the designated position. After laser positioning, the scalp was cut 0.5 cm, the skull drill was set to limit the depth as planned, and the hole was drilled through the navigation channel. The long-handled monopolar electro-cutting dura mater and electro-coagulation of brain tissue were performed to reduce bleeding, and the drainage tube was placed. The drainage tube was gradually inserted into the hematoma cavity by following the channel until it reached the target position. The drainage tube was shallow for 2 cm under the skin and fixed, and the scalp was sutured all over.

### Operation-related indexes:

The intraoperative blood loss, operation time, postoperative awake time and hematoma clearance rate of the two groups were compared and analyzed. Calculation method of hematoma clearance rate: Within 24 h after the operation, a follow-up head CT scan was conducted to measure the preoperative hematoma volume and postoperative residual hematoma volume. The difference was residual hematoma volume, and hematoma clearance rate = residual hematoma volume/preoperative hematoma volume × 100%.

### Comparative analysis of the incidence of postoperative complications:

including the incidence of urinary infection, intracranial infection, rebleeding, deep venous thrombosis, etc.

### Comparative analysis of inflammatory factors:

Prior to therapy and one week after therapy, venous blood was picked from patients, and the degrees of changes in inflammatory factors such as tumor necrosis factor (TNF-a), interleukin 6 (IL-6), and C-reactive protein (CRP) were gauged through enzyme-linked immunosorbent assay (ELISA)

### Comparison of the Glasgow Coma Scale (GCS) before and one week after the operation between the two groups:

The GCS includes three items: motor response (1-6 points), eye opening (1-4 points), and verbal response (1-5 points), with a total score of 3-15 points. The lower the score, the more severe the coma of patients.^9^

### Comparative analysis of neurological function recovery:

The neurological function of both groups was assessed before and one week after the operation using the National Institutes of Health Stroke Scale (NIHSS) score, which has a total score range of 0 to 42. A higher score indicates more severe neurological damage in patients.^10^ The two groups of patients were followed up for one month.

### Statistical analysis:

The statistical analysis of all data in this study was conducted using SPSS 20.0 software. The enumerated data were expressed via n (%). The measurement data were presented as (*χ̅*±*S*). The analysis of inter-group data was performed using the Two Independent Samples t-test, while the analysis of intra-group data was done using the Paired t-test. The comparison between the two groups was conducted using the χ² test. A statistically meaningful contrast was considered when p<0.05.

## RESULTS

The study included a total of 120 patients, with 60 in the experimental group and 60 in the control group. In Table-I, there was no meaningful contrast in the general data between the two groups, indicating that the groups were comparable.

**Table-I:** A comparative analysis on the basic information between the control and experimental groups (*χ̅*±*S*) n=60.

Indexes	Experimental group	Control group	t/χ^2^	P
Age (years old)	65.37±4.51	66.28±4.08	1.17	0.25*
Male (cases, %)	33 (55%)	32 (53%)	0.03	0.86^△^
Bleeding site				
Basal ganglia	35 (58%)	37 (62%)	0.14	0.71^△^
Thalamus	7 (12%)	5 (8%)	0.37	0.54^△^
Ventricles	6 (10%)	8 (13%)	0.32	0.57^△^
Lobe	12 (20%)	10 (17%)	0.22	0.64^△^
BMI (kg/m^2^)	23.62±3.21	23.38±3.46	0.38	0.70*
Bleeding volume (ml)	43.18±8.06	42.82±7.63	0.26	0.80*
Onset time (h)	12.36±3.42	12.74±4.08	0.56	0.58*
GCS score	10.23±4.32	10.48±4.50	0.31	0.76*

***Note:***

* independent-sample t-test;

△ χ^2^ test.

There was a meaningful distinction in operation’s length between the two groups, with the experimental possessing a briefer operation time (p=0.00). Additionally, the experimental group possessed a considerably more prominent blood drainage volume throughout the operation and a more excellent hematoma clearance rate in comparison with the control group (p=0.00). There was a statistically meaningful contrast (p=0.02) in the postoperative awake time between the experimental and control groups, with the former having a significantly shorter awake time. Table-II.

**Table-II:** A comparative analysis of operation-related indices between the two groups (*χ̅*±*S*) n=60.

Group	Length of operation (h)	Blood drainage volume (ml)	Postoperative awake time (h)	Hematoma clearance rate (%)
Experimental group	1.03±0.32	33.17±8.68	20.60±9.72	90.37±7.38
Control group	1.78±0.51	19.46±7.24	24.38±8.63	66.41±7.05
*t*	9.75	9.40	2.26	18.18
*P* *	<0.001	<0.001	0.03	<0.001

***Note:*** p<0.05,

* independent-sample t-test.

There was a statistically considerable distinction (p=0.03) in the incidence of postoperative complications between the experimental and control groups. Table-III. The incidence of complications in the experimental group was lower (10%) compared to the control group (25%), demonstrating a significant decrease.

**Table-III:** A comparative analysis conducted to evaluate the incidence of postoperative complications between the two groups (*χ̅*±*S*) n=60.

Group	Intracranial infection	Urinary infection	Deep venous thrombosis	Rebleeding	Incidence
Experimental group	0	3	3	0	6 (10%)
Control group	5	3	3	4	15 (25%)
χ^2^					4.68
P^△^					0.03

***Note:***

△ χ^2^ test, p<0.05.

Prior to therapy, there were no statistically substantial distinctions (p>0.05) in IL-6, TNF-a, CRP, and other indexes between the experimental and control groups. After therapy shows in Table-IV, the experimental group exhibited a considerable reduction in CRP, TNF-a, and IL-6 indexes in comparison with the control group, with statistically considerable distinctions (p=0.00).

**Table-IV:** A comparative analysis performed to assess the changes in inflammatory factors between the two groups prior to and later than treatment (*χ̅*±*S*) n=60.

Indexes		Experimental group	Control group	t	p
TNF-ɑ (ng/L)	Prior to therapy	23.56±8.20	24.12±8.73	0.36	0.72^△^
	Later than therapy*	8.73±2.01	10.37±2.21	4.26	<0.001^△^
CRP (mg/L)	Prior to therapy	34.16±8.43	34.63±7.98	0.31	0.75^△^
	Later than therapy*	7.52±2.05	9.29±3.48	3.39	0.001^△^
IL-6 (ng/L)	Prior to therapy	9.25±1.63	9.30±1.58	0.17	0.87^△^
	Later than therapy*	3.44±1.23	5.08±1.26	7.21	<0.001^△^

***Notes:***

△ independent-sample t-test;

* Paired t-test, p<0.05 compared with this group prior to therapy.

There were no statistically substantial contrasts (p>0.05) in motor response, eye opening, and verbal response prior to therapy between the experimental and control groups. It can be seen from Table-V that after treatment, the experimental group showed considerably more prominent indexes in motor response, eye opening, and verbal response compared to the control group, with statistically significant differences (p=0.00). Prior to therapy, there were no statistically substantial contrasts (p>0.05) in NHISS grades between the experimental and control groups. Both groups, however, revealed a considerable reduction in NHISS scores after therapy compared to prior to treatment (p=0.00). The experimental group possessed a substantially more tremendous diminution in NHISS grades in comparison with the control group, with a statistically substantial contrast (p=0.00). Table-VI

**Table-V:** Comparative analysis of long-term therapeutic effects of the two groups (*χ̅*±*S*) n=60.

Indexes		Experimental group	Control group	t	p
Motor response	Prior to therapy	3.87±0.43	3.93±0.41	0.87	0.39^△^
	Later than therapy*	5.68±0.50	5.10±0.30	7.69	<0.001^△^
Eye opening	Prior to therapy	2.17±0.46	2.23±0.43	0.83	0.41
	Later than therapy*	3.72±0.45	3.20±0.40	6.59	<0.001^△^
Verbal response	Prior to therapy	3.25±0.63	3.30±0.53	0.47	0.64^△^
	Later than therapy*	4.65±0.48	4.28±0.45	4.29	<0.001^△^

***Notes:***

△ independent-sample t-test;

* Paired t-test, p<0.05 compared with this group prior to therapy.

**Table-VI:** A comparative analysis conducted to evaluate the changes in NHISS scores between the two groups prior to and after treatment (*χ̅*±*S*) n=60.

Group	Prior to therapy	Later than therapy*	t	p
Experimental group*	22.83±2.32	11.25±2.43	31.37	<0.001*
Control group*	22.68±2.06	14.73±3.04	21.66	<0.001*
t	0.38	6.94		
P	0.71^△^	<0.001^△^		

***Notes:***

△ independent-sample t-test;

* Paired t-test, *p<0.05 compared with this group prior to therapy.

## DISCUSSION

Our study verified that the robot-assisted group had a considerably shorter operation duration than the control group(p=0.00). Additionally, the volume of intraoperative blood drainage and hematoma clearance rate were considerably more eminent in the robot-assisted group than the control group (p=0.00). However, the postoperative awake time was meaningfully shorter in the robot-assisted group in comparison the control group (p=0.02). In comparison with the conventional group, the prevalence of complications was considerably more down in the experimental group (p=0.03). Post-treatment indicators, which covers TNF-a, CRP, and IL-6 were meaningfully reduced (p=0.00), while GCS grades showed a significant increase, and NHISS scores demonstrated a meaningful reduction (p=0.00) in the experimental group in comparison with the control group (p=0.00).

In the acute stage, surgery is the preferred therapy for patients who have hypertensive intracerebral hemorrhage. When surgical treatment is performed, the intracerebral hematoma can be removed as early as possible to reduce intracranial pressure, the mechanical compression of the surrounding brain tissue by the hematoma can be relieved, the chemical damage to the brain tissue by toxic substances produced by hematoma decomposition can be reduced, and secondary brain edema and secondary brain damage can be reduced. In addition, it can ameliorate cerebral ischemia and hypoxia, restore compressed neurons, break the vicious circle that endangers the life safety of patients, reduce the disability rate and mortality rate of patients, and improve the quality of life.^11^

In this regard, craniotomy for hematoma removal can quickly remove a hematoma, reduce intracranial pressure, control the site of active bleeding and reduce the incidence of rebleeding. However, since the large trauma, it does not succeed in achieving the objective of reducing mortality rates and enhancing the quality of life for those who survive. Therefore, except for the application in the treatment of patients with combined brain herniation, other methods have been replaced by minimally invasive surgical methods. Currently, the commonly used minimally invasive surgery mainly consists of endoscopic surgery, minimally invasive puncture intracranial hematoma removal through hard channels, stereotactic surgery, etc.^12^ With the advantages of simple operation, strong practicability, short operation time and little injury, stereotactic puncture hematoma removal is adopted by most clinicians.

In recent years, neurosurgery robot has been applied in the clinic, providing a stable guarantee for accurate puncture surgery. The robot is composed of three parts: a computer software system, a locator and a robotic arm, with the first being the robot’s “brain”. After importing the patient’s relevant CT or CTA, MR and other data into the software system, the doctor can carry out three-dimensional reconstruction, observe the multi-modal image of the head, and plan the best surgical puncture path before the operation from multiple angles and paths, so as to avoid blood vessels and functional areas as much as possible. As the “eyes” of the robot, the locator is used to identify the MARK on the patient’s head and carry out calibration registration. Before the final puncture, the verification point is verified to ensure positioning accuracy. Also, real-time tracking is conducted and the robotic arm is ensured to move along the planned path to the planned surgical position.

The robotic arm is the “hand” of the robot. After a series of planning, verification and calibration, the robotic arm can accurately locate the surgical puncture position planned by the doctor, including the puncture direction and puncture depth. Meanwhile, the robotic arm can serve as a multifunctional surgical operation platform to minimize puncture error.^13^ Doctors can provide a novel treatment method for intracerebral hemorrhage with the help of robots, taking advantage of their minimally invasive characteristics, precision, high safety and selection of optimal puncture path.^6^ Specifically, neurosurgical robots, with the outstanding advantage of being suitable for performing high-precision operations, have brought about a series of technological changes in terms of precise positioning, minimal injury and quality of operation. Based on the development trend of pursuing safety, minimally invasive and precision in neurosurgery, robots have also incomparable good results in minimally invasive treatment on this basis.^14^

According to a retrospective study carried out by Yao et al.,^15^ robot-assisted surgery is characterized by superior breakthrough points, accuracy, and efficiency. The neurosurgical robot’s navigation and positioning system has the added benefits of minimal trauma and high accuracy, making it ideal for surgical planning based on the shape of the hematoma. Therefore, it is well-suited for the drainage of cerebral hemorrhage and evacuation of hematoma. Wang et al.’s 16 retrospective analysis revealed that robot-assisted stereotactic technology can guide hematoma puncture with exceptional accuracy, with an average positioning error of 1.28±0.49 mm and an average drainage duration of 3.4 days. A three-month follow-up after the surgery demonstrated that all patients experienced an improvement in neurological function and quality of life. No intracranial infection occurred, and the 30-day mortality rate was more reduced than that of the conventional puncture group (2.3% and 9.2%, respectively).

The statistical significance between the two groups is noteworthy(p<0.01). Additionally, Wu et al.^17^ proposed that patients who underwent robot-assisted punctures for six months exhibited improved neurological function outcomes and a diminished inflammatory response. Perihematomal inflammatory edema following cerebral hemorrhage is a crucial factor associated with unfavorable prognostic outcomes.^18^ Consequently, this approach is not only safer but also more efficient. The combination of robot-assisted neuroendoscopic hematoma removal and ICP monitoring may enhance the clinical outcome and treatment results for patients with HICH. Xiong et al.’s study^19^ corroborated that the robot group had a shorter operation and drainage duration, a higher degree of hematoma evacuation, improved neurological function, and fewer complications.

### Limitations:

It includes a short follow-up period and small sample size, as it was conducted at a single center. In future clinical research, it is imperative to expand the sample size, prolong the follow-up period, and make more the comparative analysis of patients with varying onset time windows to provide a more comprehensive understanding of the long-term and short-term effects of this treatment method.

## CONCLUSIONS

The utilization of stereotactic robot-guided puncture for hematoma drainage has demonstrated a favorable clinical therapeutic outcome in managing cerebral hemorrhage, accompanied by reduced, light inflammatory reaction, high efficiency in removing hematoma, good recovery of neurological function and higher surgical safety. It is a reliable surgical method for treating cerebral hemorrhage.

### Authors’ Contributions:

**XG** and **HD:** Carried out the studies, participated in collecting data, drafted the manuscript, are responsible and accountable for the accuracy or integrity of the work

**XL:** Performed the statistical analysis and participated in its design.

**ZL:** Participated in acquisition, analysis, or interpretation of data and drafting the manuscript.

All authors have read and approved the final manuscript.

## References

[1] Gross BA, Jankowitz BT, Friedlander RM (2019). Cerebral Intraparenchymal Hemorrhage:A Review. JAMA.

[2] Garg R, Biller J (2019). Recent advances in spontaneous intracerebral hemorrhage. F1000Res.

[3] Schrag M, Kirshner H (2020). Management of Intracerebral Hemorrhage:JACC Focus Seminar. J Am Coll Cardiol.

[4] Hostettler IC, Seiffge DJ, Werring DJ (2019). Intracerebral hemorrhage:an update on diagnosis and treatment. Expert Rev Neurother.

[5] Deng C, Ji Y, Song W, Bi J (2022). Clinical effect of minimally invasive aspiration and drainage of intracranial hematoma in the treatment of cerebral hemorrhage. Pak J Med Sci.

[6] Bhatia K, Hepburn M, Ziu E, Siddiq F, Qureshi AI (2018). Modern Approaches to Evacuating Intracerebral Hemorrhage. Curr Cardiol Rep.

[7] Jin P, Jiang W, Bao Q, Wei W, Jiang W (2022). Predictive nomogram for soft robotic hand rehabilitation of patients with intracerebral hemorrhage. BMC Neurol.

[8] Magid-Bernstein J, Girard R, Polster S, Srinath A, Romanos S, Awad IA (2022). Cerebral Hemorrhage:Pathophysiology, Treatment, and Future Directions. Circ Res.

[9] Cook NF (2021). The Glasgow Coma Scale:A European and Global Perspective on Enhancing Practice. Crit Care Nurs Clin North Am.

[10] Chalos V, Van der Ende NAM, Lingsma HF, Mulder MJHL, Venema E, Dijkland SA (2020). National Institutes of Health Stroke Scale:An Alternative Primary Outcome Measure for Trials of Acute Treatment for Ischemic Stroke. Stroke.

[11] Vitt JR, Sun CH, Le Roux PD, Hemphill JC (2020). Minimally invasive surgery for intracerebral hemorrhage. Curr Opin Crit Care.

[12] Yuan H, Feng J, Lin X, Li S (2022). The effect of early vs. late CT-guided stereotactic hematoma aspiration on neurological function recovery in patients with hypertensive cerebral hemorrhage in the basal ganglia:a retrospective comparative cohort study. Ann Palliat Med.

[13] Chen Y, Godage IS, Sengupta S, Liu CL, Weaver KD, Barth EJ (2019). MR-conditional steerable needle robot for intracerebral hemorrhage removal. Int J Comput Assist Radiol Surg.

[14] Musa MJ, Carpenter AB, Kellner C, Sigounas D, Godage I, Sengupta S (2022). Minimally Invasive Intracerebral Hemorrhage Evacuation:A review. Ann Biomed Eng.

[15] Yao Y, Hu W, Zhang C, Wang X, Zheng Z, Sang L (2023). A comparison between robot-guided and stereotactic frame-based stereoelectroencephalography (SEEG) electrode implantation for drug-resistant epilepsy. J Robot Surg.

[16] Wang T, Zhao QJ, Gu JW, Shi TJ, Yuan X, Wang J (2019). Neurosurgery medical robot Remebot for the treatment of 17 patients with hypertensive intracerebral hemorrhage. Int J Med Robot.

[17] Wu S, Wang H, Wang J, Hu F, Jiang W, Lei T (2021). Effect of Robot-Assisted Neuroendoscopic Hematoma Evacuation Combined Intracranial Pressure Monitoring for the Treatment of Hypertensive Intracerebral Hemorrhage. Front Neurol.

[18] Al-Kawaz MN, Hanley DF, Ziai W (2020). Advances in Therapeutic Approaches for Spontaneous Intracerebral Hemorrhage. Neurotherapeutics.

[19] Xiong R, Li F, Chen X (2020). Robot-assisted neurosurgery versus conventional treatment for intracerebral hemorrhage:A systematic review and meta-analysis. J Clin Neurosci.

